# Near-infrared spectroscopy during stagnant ischemia estimates central venous oxygen saturation and mixed venous oxygen saturation discrepancy in patients with severe left heart failure and additional sepsis/septic shock

**DOI:** 10.1186/cc8929

**Published:** 2010-03-23

**Authors:** Hugo Možina, Matej Podbregar

**Affiliations:** 1 Clinical Department of Intensive Care Medicine, University Clinical Centre Ljubljana, Zaloska cesta 7, SI-1000 Ljubljana, Slovenia

## Abstract

**Introduction:**

Discrepancies of 5-24% between superior vena cava oxygen saturation (ScvO_2_) and mixed venous oxygen saturation (SvO_2_) have been reported in patients with severe heart failure. Thenar muscle tissue oxygenation (StO_2_) measured with near-infrared spectroscopy (NIRS) during arterial occlusion testing decreases slower in sepsis/septic shock patients (lower StO_2 _deoxygenation rate). The StO_2 _deoxygenation rate is influenced by dobutamine. The aim of this study was to determine the relationship between the StO_2 _deoxygenation rate and the ScvO_2_-SvO_2 _discrepancy in patients with severe left heart failure and additional sepsis/septic shock treated with or without dobutamine.

**Methods:**

Fifty-two patients with severe left heart failure due to primary heart disease with additional severe sepsis/septic shock were included. SvO_2 _and ScvO_2 _were compared to the thenar muscle StO_2 _before and during arterial occlusion.

**Results:**

SvO_2 _correlated significantly with ScvO_2 _(Pearson correlation 0.659, *P *= 0.001), however, Bland Altman analysis showed a clinically important difference between both variables (ScvO_2_-SvO_2 _mean 72 ± 8%, ScvO_2_-SvO_2 _difference 9.4 ± 7.5%). The ScvO_2_-SvO_2 _difference correlated with plasma lactate (Pearson correlation 0.400, *P *= 0.003) and the StO_2 _deoxygenation rate (Pearson correlation 0.651, *P *= 0.001). In the group of patients treated with dobutamine, the ScvO_2_-SvO_2 _difference correlated with plasma lactate (Pearson correlation 0.389, *P *= 0.011) and the StO_2 _deoxygenation rate (Pearson correlation 0.777, *P *= 0.0001).

**Conclusions:**

In patients with severe heart failure with additional severe sepsis/septic shock the ScvO_2_-SvO_2 _discrepancy presents a clinical problem. In these patients the skeletal muscle StO_2 _deoxygenation rate is inversely proportional to the difference between ScvO_2 _and SvO_2_; dobutamine does not influence this relationship. When using ScvO_2 _as a treatment goal, the NIRS measurement may prove to be a useful non-invasive diagnostic test to uncover patients with a normal ScvO_2 _but potentially an abnormally low SvO_2_.

**Trial Registration:**

NCT00384644 ClinicalTrials.Gov.

## Introduction

Maintenance of adequate oxygen delivery (DO_2_) is essential to preserve organ function, because a sustained low DO_2 _leads to organ failure and death [1]. Low cardiac output states (cardiogenic, hypovolemic and obstructive types of shock), anemic and hypoxic hypoxemia are characterized by a decreased DO_2 _but a preserved oxygen extraction ratio. In distributive shock, the oxygen extraction capability is altered so that the critical oxygen extraction ratio is typically decreased [2]. Measurement of mixed venous oxygen saturation (SvO_2_) from the pulmonary artery is used for calculations of oxygen consumption and has been advocated as an indirect index of tissue oxygenation and a prognostic predictor in critically ill patients [3-6]. However, catheterization of the pulmonary artery is costly, has inherent risks and its usefulness remains under debate [7,8].

Not surprisingly the monitoring of central venous oxygen saturation (ScvO_2_) was suggested as a simpler and cheaper assessment of global DO_2 _to oxygen consumption ratio [1,2].

A concern with ScvO_2 _compared with mixed venous oxygen saturation (SvO_2_) is that it may not accurately reflect global hypoxia, because organs with capillary beds that drain into the inferior vena cava or coronary sinus will not be involved in this measurement. Healthy resting individuals have a ScvO_2 _that is slightly lower than the SvO_2 _[3]. In heart failure and shock, however, this situation is reversed. Most authors attribute this pattern to changes in the distribution of cardiac output that occur in periods of haemodynamic instability. In shock states, blood flow to the splanchnic and renal circulations fall, while flow to the heart and brain is maintained due to redistribution of blood away from the mesenteric and renal vascular beds and additional right heart dysfunction [4]. Discrepancies of 5 to 24% have been reported [5-7,9].

Near infrared spectroscopy (NIRS) is a technique used for continuous, non-invasive, bedside monitoring of tissue oxygen saturation (StO_2_) [8,10].

We have previously shown that skeletal muscle StO_2 _does not estimate SvO_2 _in patients with severe left heart failure and additional severe sepsis or septic shock. However, in patients with severe left heart failure without additional severe sepsis or septic shock, StO_2 _values could be used for fast noninvasive SvO_2 _estimation; the trend of StO_2 _may be substituted for the trend of SvO_2 _[8].

We have also shown that thenar skeletal muscle StO_2 _during stagnant ischemia (deoxygenation rate during arterial occlusion test) decreases slower in septic shock patients compared with patients with severe sepsis or localized infection or healthy volunteers [10].

Impaired skeletal muscle microcirculation, especially impaired deoxygenation rate during arterial occlusion test, was recently detected in patients with chronic heart failure. Dobutamine, but not levosimendan, partially reversed this impairment [11].

The aim of current study was to combine our previous findings. We tested the hypothesis that in patients with severe left heart failure and additional sepsis/septic shock the skeletal muscle deoxygenation rate during an arterial occlusion test could predict a ScvO_2_-SvO_2 _discrepancy. The second aim was to explore the effect of dobutamine treatment on any ScvO_2_-SvO_2 _discrepancy.

## Materials and methods

### Patients

The study protocol was approved by the National Ethics Committee of Slovenia; informed consent was obtained from all patients or their relatives. The study was performed between October 2004 and June 2007.

After initial hemodynamic resuscitation according to early goal-directed therapy [12] and Surviving Sepsis Campaign guidelines [13], transthoracic echocardiography for the assessment of left ventricular volume, ejection fraction (Simpson's rule) and valvular function was performed in all patients admitted to our ICU (Hewlett-Packard HD 5000, Hewlett Packard, Andover, MA, USA) by experienced ICU doctors (HM and MP) trained in echocardiography.

In patients with primary heart disease, low cardiac output, and no signs of hypovolemia, a right heart catheterization with a pulmonary artery floating catheter (Swan-Ganz CCOmboV CCO/SvO_2_/CEDV, Edwards Lifesciences, Irvine, CA, USA) was performed following a decision of the treating physician. The site of insertion was confirmed by the transducer waveform, the length of catheter insertion, and chest radiography. Systemic arterial pressure was measured invasively using radial or femoral arterial catheterization. Consecutive patients with severe left heart failure due to primary heart disease (left ventricular systolic ejection fraction below 40%, pulmonary artery occlusion pressure above 18 mmHg) and additional severe sepsis/septic shock were included in our study. Severe sepsis and septic shock were defined according to the 1992 American College of Chest Physicians/Society of Critical Care Medicine (ACCP/SCCM) consensus conference definitions [14]. Patients with heart failure confirmed by echocardiography without sepsis/septic shock were excluded. Patients with cachexia were not included.

Patients were divided into two groups depending on treatment with dobutamine or not.

All patients received standard treatment of localized infection, severe sepsis and septic or cardiogenic shock including: source control, fluid infusion, catecholamine infusion, organ failure replacement and/or support therapy, intensive control of blood glucose and corticosteroid substitution therapy according to current Surviving Sepsis Campaign Guidelines [13]. Mechanically ventilated patients were sedated with midazolam and/or propofol infusion. Paralytic agents were not used.

### Measurements

#### Skeletal muscle oxygenation

Thenar muscle StO_2 _was measured non-invasively by NIRS (25 mm Probe, InSpectra™, Hutchinson Technology Inc., West Highland Park Drive NE, MN, USA) [8,10,15]. Maximal thenar muscle StO_2 _was located by moving the probe over the thenar prominence. StO_2 _was continuously monitored and stored onto a computer using InSpectra™ software. The average of StO_2 _changing over a 15 second span was used. The arterial occlusion test was performed as previously reported [10]: StO_2 _was monitored before and during (StO_2 _deoxygenation rate) upper limb ischemia until StO_2 _decreased to 40%. Upper limb ischemia was induced by rapid automatic pneumatic cuff inflation (to 260 mmHg) placed above the elbow.

#### Severity of disease

Sepsis-related Organ Failure Assessment (SOFA) score was calculated at the time of each measurement to assess the level of organ dysfunction [16]. Dobutamine and norepinephrine requirement represented the dose of drug during the StO_2 _measurement. Use of an intra-aortic balloon pump during the ICU stay is reported.

Plasma lactate concentration was measured using an enzymatic colorimetric method (Lactate, Roche Diagnostics, Hoffman-La Roche, Basel, Switzerland) at the time of each StO_2 _measurement.

#### Laboratory analysis

Blood was withdrawn from the superior vena cava approximately 2 cm above the right atrium and from the pulmonary artery at the time of each StO_2 _measurement to determine ScvO_2 _(%) and SvO_2 _(%), respectively. In view of known problems arising during sampling from the pulmonary artery, including the possibility of contaminating arterial blood with pulmonary capillary blood, all samples from this site were withdrawn over 30 seconds, using a low-negative pressure technique, without inflating the balloon. A standard volume of 1 mL of blood was obtained from each side after withdrawal of dead-space blood and flushing fluid. All measurements were made using a cooximeter (RapidLab 1265, Bayer HealthCare, Leverkusen, Germany).

### Data analysis

A sample size of 41 patients was estimated for a correlation coefficient of 0.6 with a desired power o f0.95 and alpha of 0.01 (SigmaPlot 2004 for Windows, version 9.01 SyStat Software, Inc., Chicago, IL, USA).

Data was expressed as mean ± standard deviation (SD). The Mann Whitney non-parametric test was used to compare groups. A *P *value of less than 0.05 was considered statistically significant. The Pearson correlation test was applied to determine correlation (SPSS 10.0 for Windows™, SPSS Inc., Chicago, IL, USA). In order to compare ScvO_2 _and SvO_2 _we calculated bias, systemic disagreement between measurements (mean difference between two measurements), precision and the random error in measuring (SD of bias) [17]. The 95% limits of agreement were arbitrarily set following Bland and Altman as the bias ± two SD.

## Results

During the study period (20 months), 2,121 patients were admitted to the 15-bed university center internal medicine ICU. In that period 151 right heart catheterizations were performed. The final sample of 52 patients was reached after exclusion of 65 patients with heart failure without sepsis/septic shock, 24 patients who did not have heart failure, 2 patients for whom consent was not given and 8 patients for whom NIRS measurements were not performed. The detailed description of our selected population is given in Table 1. Patients were all mechanically ventilated.

**Table 1:** Description of patients

Parameter	All (n = 52)	Treatment with dobutemine (n = 43)	Treatment without dobutamine (n = 9)	*P *value
Age (years)	68 ± 13	68 ± 14	69 ± 8	0.8
Female (n)	7	5	2	0.6
Heart disease				
Ischemic heart disease (n)	42	36	6	0.4
Aortic stenosis (n)	6	4	2	0.6
Dilated cardiomyopathy (n)	1	1	0	0.9
Myocarditis (n)	3	2	1	0.6
Echocardiography				
LVEF (%)	28 ± 5	25 ± 8	29 ± 9	0.1
LVEDD (cm)	5.8 ± 0.9	5.8 ± 0.7	6.0 ± 0.9	0.2
Severe mitral regurgitation (n)	26	22	4	0.8
Cause of infection				
Pneumonia (n)	45	38	7	0.6
Urosepsis (n)	5	4	1	0.9
Other (n)	2	1	1	0.7
SOFA score	12.2 ± 2.5	12. ± 2.2	12.6 ± 2.6	0.8
ICU stay (days)	9 ± 4	9 ± 6	9 ± 5	0.9
ICU survival (%)	48	47	55	0.8

LVEF, left ventricular ejection fraction; LVEDD, left ventricular end-diastolic diameter; SOFA, Sequential Organ Failure Assessment.

Intra-aortic balloon pumps were inserted in patients who were treated with percutaneous coronary intervention and stent implantation after primary cardiac arrest due to ST-elevation myocardial infarction (STEMI; n = 42) and cardiogenic shock. Patients with STEMI after cardiac arrest were treated with medically induced hypothermia for 24 hours. During the ICU stay and before study inclusion they all developed pneumonia. All other patients were admitted to the ICU primarily because of sepsis or septic shock.

Forty-three patients were treated with dobutamine. There was no difference between patients treated with or without dobutamine in additional hemodynamic support (Table 2). Patients treated with dobutamine had a lower cardiac index (Table 3) and a higher procalcitonin value (Table 4).

**Table 2:** Treatment of patients

Treatment	All (n = 52)	Treatment with dobutemine (n = 43)	Treatment without dobutamine (n = 9)	*P *value
Norepinephrine (mg/h, n)	0.09 ± 0.10 (43)	0.08 ± 0.11 (37)	0.04 ± 0.06 (9)	0.1
Dobutamine (μg/kg/min)	-	0.47 ± 0.25	-	-
Levosimendan (n)	23	17	6	0.2
IAPB (n)	20	15	5	0.3
Mechanical ventilation(n)	52	43	9	1.0
FiO_2_	0.72 ± 0.22	0.73 ± 0.23	0.71 ± 0.23	0.8

FiO_2_, fractional inspired oxygen; IAPB, intra-aortic balloon pump.

**Table 3:** Hemodynamic data in patients with heart failure and additional sepsis treated with and without dobutamine

Hemodynamic data	All (n = 52)	Treatment with dobutemine (n = 43)	Treatment without dobutamine (n = 9)	*P *value
HR (bpm)	113 ± 20	113 ± 20	114 ± 21	0.8
SAP (mmHg)	118 ± 21	117 ± 20	124 ± 27	0.9
DAP (mmHg)	74 ± 22	76 ± 22	66 ± 21	0.4
PAP_s _(mmHg)	57 ± 14	56 ± 13	57 ± 16	0.9
PAP_d _(mmHg)	28 ± 8	27 ± 8	29 ± 7	0.4
CVP (mmHg)	16 ± 5	16 ± 5	15 ± 5	0.8
DO_2 _(ml/kg/min)	406 ± 128	391 ± 134	470 ± 121	0.1
VO_2 _(ml/kg/min)	118 ± 42	116 ± 43	126 ± 38	0.5
PAOP (mmHg)	23 ± 7	24 ± 7	22 ± 8	0.7
CI (L/min/m^2^)	2.5 ± 0.7	**2.4 ± 0.7**	**2.9 ± 0.6**	**0.03**
SvO_2 _(%)	67 ± 10%	66 ± 10	71 ± 7	0.2
ScvO_2 _(%)	77 ± 8%	77 ± 7	78 ± 10	0.6

**Bold**: statistically significant difference, *P *< 0.05.

CI, cardiac index; CVP, central venous pressure; DAP, diastolic arterial pressure; DO_2_, delivery of oxygen; HR, heart rate; PAOP, pulmonary artery occlusion pressure; PAP_d_, diastolic pulmonary arterial pressure; PAP_s_, systolic pulmonary arterial pressure; SAP, systolic arterial pressure; SvO_2_, mixed venous hemoglobin saturation; ScvO_2_, central venous oxygen saturation; VO_2_, oxygen consumption.

**Table 4:** Laboratory data

Laboratory data	All (n = 52)	Treatment with dobutemine (n = 43)	Treatment without dobutamine (n = 9)	*P *value
Core temperature (°C)	38.0 ± 0.9	37.9 ± 0.87	38.2 ± 0.92	0.5
Lactate (mmol/l)	3.5 ± 3.0	3.6 ± 3.3	3.0 ± 1.7	0.4
CRP (mg/l)	127 ± 78	124 ± 65	154 ± 120	0.6
PCT (mg/l)	6.2 ± 6.1	**7.2 ± 6.3**	**2.5 ± 4.2**	**0.01**
Leucocytes (*10^9^/l)	14.0 ± 5.4	13.8 ± 5.3	15.4 ± 6.3	0.5
Hemoglobin (g/L)	11.6 ± 1.5	11.6 ± 1.6	11.6 ± 1.0	0.9
Creatinine	198 ± 160	162 ± 142	231 ± 182	0.1
Sodium (mmol/L)	144 ± 12	144 ± 11	147 ± 14	0.8
Arterial blood gal analysis				
pH	7.35 ± 0.09	7.35 ± 0.08	7.33 ± 0.09	0.6
pCO_2 _(kPa)	4.7 ± 1.0	4.6 ± 1.0	5.3 ± 0.8	0.06
pO_2 _(kPa)	15.3 ± 5.4	14.6 ± 4.8	18.5 ± 7.4	0.1
HCO_3 _(mmol/L)	20.6 ± 5.6	20.4 ± 6.1	21.5 ± 3.9	0.5
BE(mEq/l)	-5.1 ± 6.4	-5.4 ± 6.9	-4.2 ± 4.8	0.5
SatHbO_2 _(%)	97 ± 3%	97 ± 2	98 ± 3	0.4

**Bold**: statistically significant difference, *P *< 0.05.

BE, base excess; CRP, C-reactive protein; HCO_3_, bicarbonate; PCT, procalcitonin; pCO_2_, partial pressure of carbon dioxide; pO_2_, partial pressure of oxygen; SatHbO_2_, hemoglobin oxygen saturation.

Thenar StO_2 _before (basal StO_2_) and during the vascular occlusion test is presented in Table 5. There was no difference between patients treated with and without dobutamine in NIRS data.

**Table 5:** NIRS data of skeletal muscle tissue oxygenation (StO_2_) during vascular occlusion test in patients with heart failure and additional sepsis

NIRS data	All (n = 52)	Treatment with dobutemine (n = 43)	Treatment without dobutamine (n = 9)	*P *value
Basal StO_2 _(%)	89 ± 8	88 ± 8	92 ± 6	0.1
StO_2 _deoxygenation rate (%/min)	-12.6 ± 4.9	-12.7 ± 5.2	-12.6 ± 4.6	0.9

NIRS, near-infrared spectroscopy; StO_2, _skeletal muscle tissue oxygenation.

**Figure 1:**
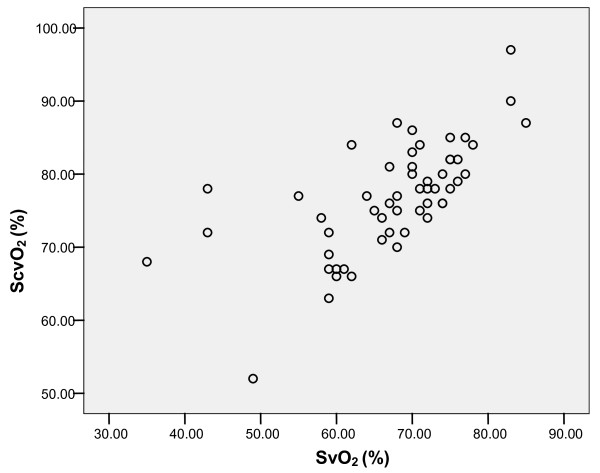
**Correlation between mixed venous (SvO_2_) and central venous saturation (ScvO_2_) in patients with heart failure and additional sepsis/septic shock**. Pearson correlation 0.659, *P *= 0.001.

**Figure 2:**
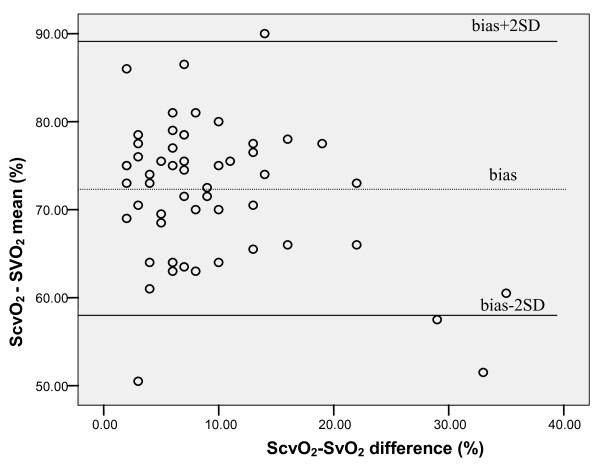
**Bland Altman analysis of clinically important difference between mixed venous (SvO_2_) and central venous saturation (ScvO_2_) in patients with heart failure and additional sepsis/septic shock**. ScvO_2_-SvO_2 _mean 72 ± 8%, Scv-Svo2 difference 9.4 ± 7.5%.

SvO_2 _correlated significantly with ScvO_2 _(Pearson correlation 0.659, *P *= 0.001; Figure 1); however, Bland Altman analysis showed a clinically important difference between both variables (ScvO_2_-SvO_2 _mean 72 ± 8%, ScvO_2_-SvO_2 _difference 9.4 ± 7.5%; Figure 2).

**Figure 3:**
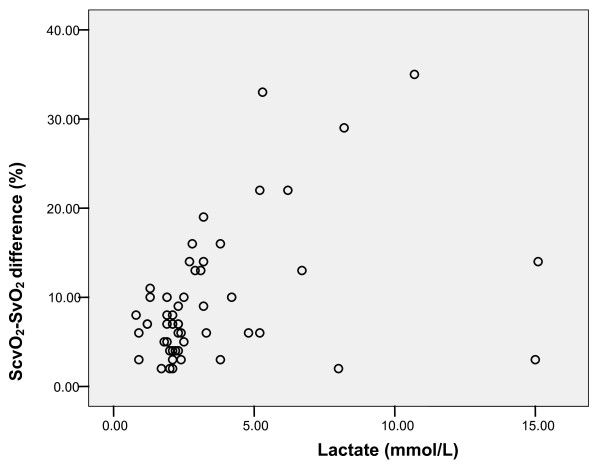
**Correlation of mixed venous (SvO_2_) and central venous saturation (ScvO_2_) difference with plasma lactate (mmol/L)**. Pearson correlation 0.400, *P *= 0.003.

**Figure 4:**
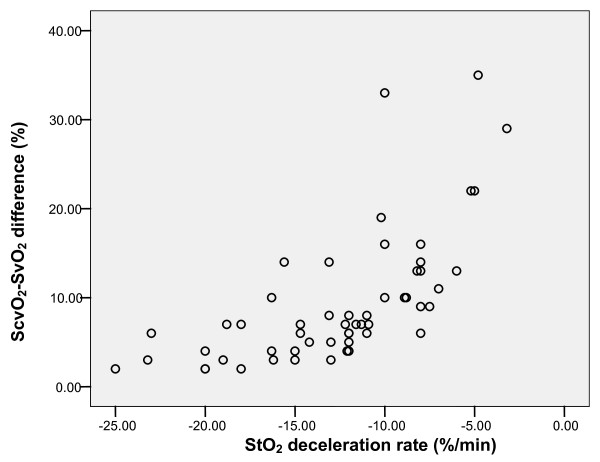
**Correlation of central venous saturation (ScvO_2_) central venous saturation (SvO_2_) difference with skeletal muscle tissue oxygenation (StO_2_) deceleration rate**. Pearson correlation 0.651, *P *= 0.001.

The ScvO_2_-SvO_2 _difference correlated with plasma lactate (Pearson correlation 0.400, *P *= 0.003; Figure 3) and StO_2 _deoxygenation rate (Pearson correlation 0.651, *P *= 0.001; Figure 4).

In the group of patients treated with dobutamine the ScvO_2_-SvO_2 _difference correlated with plasma lactate (Pearson correlation 0.389, *P *= 0.011) and StO_2 _deoxygenation rate (Pearson correlation 0.777, *P *= 0.0001).

In a small group of patients (n = 9) treated without dobutamine the ScvO_2_-SvO_2 _difference correlated with the StO_2 _deoxygenation rate (Pearson correlation 0.673, *P *= 0.033); however, there was no correlation between the ScvO_2_-SvO_2 _difference and plasma lactate (Pearson correlation 0.503, *P *= 0.139).

## Discussion

Our study confirmed the hypothesis that the skeletal muscle StO_2 _deoxygenation rate correlates (or is inversely proportional) to the ScvO_2_-SvO_2 _difference in patients with severe heart failure with additional sepsis/septic shock. This relation between the StO_2 _deoxygenation rate and the ScvO_2_-SvO_2 _difference was also present in patients treated with or without dobutamine. We also showed that these patients have a clinically considerable ScvO_2_-SvO_2 _discrepancy. Monitoring of ScvO_2 _is a simpler and cheaper assessment of global DO_2 _to oxygen consumption ratio, but its use as a treatment goal in patients with severe heart failure with additional sepsis/septic shock is questionable.

The high StO_2_/low SvO_2 _seen in patients with severe sepsis and septic shock suggests blood flow redistribution. Thenar muscle StO_2 _correlates with central venous oxygen saturation that is measured in a mixture of blood from the head and both arms [18]. In healthy resting individuals the ScvO_2 _is slightly lower than the SvO_2 _[3]. Blood in the inferior vena cava has a high oxygen content because the kidneys do not utilise much oxygen but receive a high proportion of the cardiac output [19]. Blood in the inferior vena cava blood has a higher oxygen content than blood from the upper body and the SvO_2 _is thus greater than the ScvO_2_.

This relation changes in periods of cardiovascular instability. Scheinman and colleagues performed the earliest comparison of ScvO_2 _and SvO_2 _in both hemodynamically stable and shocked patients [5]. In stable patients, ScvO_2 _was similar to SvO_2_. In patients with a failing heart, ScvO_2 _was slightly higher than SvO_2 _and in patients with shock the difference between SvO_2 _and ScvO_2 _was even more expressed (47.5% ± 15.11% vs. 58.0% ± 13.05%, respectively, *P *< 0.001). Lee and colleagues described similar findings [20]. Other more detailed studies in mixed groups of critically ill patients designed to test if the ScvO_2 _measurements could substitute the SvO_2 _showed problematically large confidence limits [6] and poor correlation between the two values [7].

Most authors attribute this pattern to changes in the distribution of cardiac output that occur in periods of hemodynamic instability. In shock states, blood flow to the splanchnic and renal circulations falls, while flow to the heart and brain is maintained [21]. This results in a fall in the oxygen content of blood in the inferior vena cava. As a consequence, in shock states the normal relation is reversed and ScvO_2 _is greater than SvO_2 _[5]. Therefore, when using ScvO_2 _or StO_2 _as a treatment goal, the relative oxygen consumption of the superior vena cava system may remain stable, while the oxidative metabolism of vital organs, such as the splanchnic region, may reach a level where a flow-limited oxygen consumption is achieved, together with a marked decrease in oxygen saturation. In this situation skeletal muscle StO_2 _provides a false favorable impression of an adequate body perfusion, because of the inability to detect organ ischemia in the lower part of the body.

In our study, three patients with septic shock had skeletal muscle StO_2 _of 75% or less (under the lower boundary of 95% confidence interval for the mean of StO_2 _in controls); they were all in septic shock (lactate value above 2.5 mmol/L) with a low cardiac index below 2.0 L/min/m^2^. These patients were probably in an early under-resuscitated phase of septic shock. The low quantity of septic patients with low StO_2 _did not allow statistical comparison of StO_2 _and SvO_2_/SvO_2 _in these types of patients. Additional research is necessary to study muscle skeletal StO_2 _in under resuscitated septic patients.

Our data are supported by previous work by Boekstegers and colleagues who measured the oxygen partial pressure distribution in bicep muscle [22]. They found low peripheral oxygen availability in cardiogenic shock compared with sepsis. In cardiogenic shock the skeletal muscle oxygen partial pressure correlated with systemic oxygen delivery (r = 0.59, *P *< 0.001) and systemic vascular resistance (r = 0.74, *P *< 0.001). No correlation was found between systemic oxygen transport variables and the skeletal muscle partial oxygen pressure in septic patients. These measurements were performed in the most common cardiovascular state of sepsis in contrast to hypodynamic shock, which is only present in the very final stage of sepsis or in patients without adequate volume replacement [23]. In a following study the same authors have shown that even in the final state of hypodynamic septic shock leading to death, the mean muscle partial oxygen pressure did not decrease to below 4.0 kPa before circulatory standstill [24].

A recent study confirmed the use of NIRS and the arterial occlusion test in the assessment of peripheral muscle microcirculation impairment in patients with congestive heart failure [11]. This impairment of microcirculation was partially reversed by infusion of the inotropic agent dobutamine but not by levosimendan. In chronic heart failure patients, dobutamine increases cardiac output and improves tissue perfusion, which leads to improvement of endothelial function and tissue oxygenation. It was demonstrated that short-term (72 hours) and short-term intermittent (for five hours, biweekly) administration of dobutamine has a sustained beneficial effect on vascular endothelial function for two weeks or longer and after four months, respectively [25,26]. Despite this effect of dobutamine on endothelial function in patients with chronic heart failure, we have not detected any difference in StO_2 _deoxygenation in our mixed population of patients with left heart failure and additional sepsis/septic shock treated with or without dobutamine. Sepsis/septic shock-related microvascular changes and the lack of inclusion of end-stage heart failure patients in our study are probably causes for discrepancy between the results of our study and the study performed by Nanas and colleagues [11].

It is known that progressive chronic heart failure leads to cardiac cachexia and decreased resting energy expenditure, both of which are worst outcome predictors [27]. Previously, we have shown that in these patients metabolism is changed to the predominant utilization of lipids [28]. However, these changes happen in stages of advanced chronic heart failure, while on the other hand in patients without cachexia the resting energy expenditure is increased proportionally to a higher New York Heart Association class [29]. No patients with cardiac cachexia were included in our study. The effects of dobutamine on skeletal muscle metabolism in patients with chronic heart failure were studied by magnetic resonance spectroscopy, which indicated that dobutamine has the ability to increase cardiac output and limb blood flow, although it does not improve oxygen delivery to the working muscle of the patients [30]. Increased resting blood flow can result in increased oxyhemoglobin content in muscle leading to increased basal StO_2 _but the StO_2 _deoxygenation rate should stay unchanged if the metabolic rate remains constant.

## Conclusions

In patients with severe heart failure with additional sepsis/septic shock, there is a clinically important discrepancy between ScvO_2 _and SvO_2_. However, with the use of arterial occlusion testing and measurement of the skeletal muscle deoxygenation rate, we can predict the ScvO_2_-SvO_2 _difference and determine adequate monitoring. Dobutamine use did not change this relation. Applying these findings in practice, in a patient with severe left heart failure, first perform arterial occlusion testing to determine the StO_2 _deoxygenation rate. If it is high (not prolonged as seen in sepsis/septic shock), estimate the SvO_2 _by using basal StO_2_. In the case of a prolonged skeletal muscle StO_2 _deoxygenation rate, look for additional sepsis, and the deoxygenation rate can estimate discrepancy between the ScvO2 and SvO_2_.

## Key messages

• In patients with severe left heart failure and additional severe sepsis or septic shock the ScvO_2_-SvO_2 _discrepancy is clinically important.

• The skeletal muscle StO_2 _deoxygenation rate estimates the ScvO_2_-SvO_2 _discrepancy in patients with severe left heart failure with additional severe sepsis or septic shock.

## Abbreviations

DO_2_: systemic oxygen delivery; NIRS: near infrared spectroscopy; SOFA: Sepsis-related Organ Failure Assessment Score; ScvO_2_: central venous oxygen saturation; SD: standard deviation; STEMI: ST-elevation myocardial infarction; StO_2_: tissue oxygen consumption; SvO_2_: mixed venous oxygen saturation.

## Competing interests

The authors declare that they have no competing interests.

## Authors' contributions

HM contributed to original observation, conception, design, acquisition of data, analysis and interpretation, and drafting the manuscript. MP contributed to conception, design, acquisition of data, analysis and interpretation, and drafting the manuscript.

## References

[B1] Rivers E Nguyen B Havstad S Ressler J Muzzin A Knoblich B Peterson E Tomlanovich M Early goal-directed therapy in the treatment of severe sepsis and septic shock N Engl J Med 2001 345 1368 1377 10.1056/NEJMoa010307 11794169

[B2] Reinhart K Kuhn HJ Hartog C Bredle DL Continuous central venous and pulmonary artery oxygen saturation monitoring in the critically ill Intensive Care Med 2004 30 1572 1578 10.1007/s00134-004-2337-y 15197435

[B3] Barratt-Boyes Bg Wood EH The oxygen saturation of blood in vena cava, right heart chambers and pulmonary vessels of healthy subjects J Lab Clin Med 1957 50 93 106 13439270

[B4] Lee J Wright F Barber R Central venous oxygen saturation in shock: a study in men Anesthesiology 1972 36 472 478 10.1097/00000542-197205000-00012 4553795

[B5] Scheinman MM Brown MA Rapaport E Critical assesment of use of central venous oxygen saturation as a mirror of mixed venous oxygen saturation in severly ill cardiac patients Circulation 1969 40 165 172 5796787 10.1161/01.cir.40.2.165

[B6] Edwards JD Mayall RM Importance of the sampling site for measurement of mixed venous oxygen saturation in shock Crit Care Med 1998 26 1356 1360 10.1097/00003246-199808000-00020 9710094

[B7] Martin C Auffray JP Badetti C Perrin G Papazian L Gouin F Monitoring of central venous oxygen saturation versus mixed venous oxygen saturation in critically ill patients Intensive Care Med 1992 18 101 104 10.1007/BF01705041 1613187

[B8] Podbregar M Mozina H Skeletal muscle oxygen saturation does not estimate mixed venous oxygen saturation in patients with severe left heart failure and additional severe sepsis or septic shock Crit Care 2007 11 R6 10.1186/cc5153 17227587 PMC2147710

[B9] Reinhart K Rudolph T Bredle DL Hannemann L Cain SM Comparison of central-venous to mixed-venous oxygen saturation during changes in oxygen supply/demand Chest 1989 95 1216 1221 10.1378/chest.95.6.1216 2721255

[B10] Pareznik R Knezevic R Voga G Podbregar M Changes in muscle tissue oxygenation during stagnant ischemia in septic patients Intensive Care Med 2006 32 87 92 10.1007/s00134-005-2841-8 16261341

[B11] Nanas S Gerovasili V Dimopoulos S Pierrakos C Kourtidou S Kaldara E Sarafoglou S Venetsanakos J Roussos C Nanas J Anastasiou-Nana M Inotropic agents improve the peripheral microcirculation of patients with end-stage chronic heart failure J Card Fail 2008 14 400 406 10.1016/j.cardfail.2008.02.001 18514932

[B12] Rivers E Nguyen B Havstad S Ressler J Muzzin A Knoblich B Peterson E Tomlanovich M Early goal-directed therapy in the treatment of severe sepsis and septic shock N Engl J Med 2001 345 1368 1377 10.1056/NEJMoa010307 11794169

[B13] Dellinger RP Carlet JM Masur H Gerlach H Calandra T Cohen J Gea-Banacloche J Keh D Marshall JC Parker MM Ramsay G Zimmerman JL Vincent JL Levy MM Surviving Sepsis Campaign guidelines for management of severe sepsis and septic shock Intensive Care Med 2004 30 536 555 10.1007/s00134-004-2398-y 14997291

[B14] Bone RC Balk RA Cerra FB Dellinger RP Fein AM Knaus WA Schein RM Sibbald WJ Definitions for sepsis and organ failure and guidelines for the use of innovative therapies in sepsis. The ACCP/SCCM Consensus Conference Committee. American College of Chest Physicians/Society of Critical Care Medicine Chest 1992 101 1644 1655 10.1378/chest.101.6.1644 1303622

[B15] Strahovnik I Podbregar M Measurment of skeletal muscle tissue oxygenation in critically ill Signa Vitae 2008 3 43 50

[B16] Vincent JL Moreno R Takala J Willatts S De Medonca A Bruining H Reinhart CK Suter PM Thijs LG The SOFA (Sepsis-related Organ Failure Assessment) score to describe organ dysfunction/failure Intensive Care Med 1996 22 707 710 10.1007/BF01709751 8844239

[B17] Bland JM Altman DG Statistical methods for assessing agreement between two methods of clinical measurements Lancet 1986 1 307 310 2868172

[B18] Mesquida J Masip J Gili G Artigas A Baigorri F Thenar oxygen saturation measured by near infrared spectroscopy as a noninvasive predictor of low central venous oxygen saturation in septic patients Intensive Care Med 2009 35 1106 1109 10.1007/s00134-009-1410-y 19183952

[B19] Cargill W Hickam J The oxygen consumption of the normal and diseased human kidney J Clin Invest 1949 28 526 532 10.1172/JCI102100 16695707 PMC439631

[B20] Lee J Wright F Barber R Stanley L Central venous oxygen saturation in shock: a study in man Anesthesiology 1972 36 472 478 10.1097/00000542-197205000-00012 4553795

[B21] Forsyth R Hoffbrand B Melmon K Re-distribution of cardiac output during hemorrhage in the unanesthetized monkey Circ Res 1970 27 311 4989130 10.1161/01.res.27.3.311

[B22] Boekstegers P Weidenhoefer St Pilz G Werdan K Peripheral oxygen availability within skeletal muscle in sepsis and septic shock: comparison to limited infection and cardiogenic shock Infection 1991 19 317 323 10.1007/BF01645355 1800370

[B23] Parker MM Parrillo JE Septic shock: hemodynamics and pathogenesis JAMA 1983 250 3324 3327 10.1001/jama.250.24.3324 6196497

[B24] Boekstegers P Weidenhoefer Kapsner T Werdan K Skeletal muscle partial pressure of oxygen in patients with sepsis Crit Care Med 1994 22 640 650 10.1097/00003246-199404000-00021 8143474

[B25] Patel MB Kaplan IV Patni RN Levy D Strom JA Shirani J LeJemtel TH Sustained improvement in flow-mediated vasodilation after short-term administration of dobutamine in patients with severe congestive heart failure Circulation 1999 99 60 64 9884380 10.1161/01.cir.99.1.60

[B26] Freimark D Feinberg MS Matezky S Hochberg N Shechter M Impact of short-term intermittent intravenous dobutamine therapy on endothelial function in patients with severe chronic heart failure Am Heart J 2004 148 878 882 10.1016/j.ahj.2004.04.013 15523321

[B27] Anker SD Ponikowski P Varney S Chua TP Clark AL Webb-Peploe KM Harrington D Kox WJ Poole-Wilson PA Coats AJ Wasting as independent risk factor for mortality in chronic heart failure Lancet 1997 349 1050 1053 10.1016/S0140-6736(96)07015-8 9107242

[B28] Podbregar M Voga G Effect of selective and nonselective beta-blockers on resting energy production rate and total body substrate utilization in chronic heart failure J Card Fail 2002 8 369 378 10.1054/jcaf.2002.130238 12528088

[B29] Obisesan TO Toth MJ Donaldson K Gottlieb SS Fisher ML Vaitekevicius P Poehlman ET Energy expenditure and symptom severity in men with heart failure Am J Cardiol 1996 77 1250 1252 10.1016/S0002-9149(96)00176-2 8651109

[B30] Mancini DM Schwartz M Ferraro N Seestedt R Chance B Wilson JR Effect of dobutamine on skeletal muscle metabolism in patients with congestive heart failure Am J Cardiol 1990 65 1121 1126 10.1016/0002-9149(90)90325-U 2330898

